# Identification of genes required for the fitness of *Streptococcus equi* subsp. *equi* in whole equine blood and hydrogen peroxide

**DOI:** 10.1099/mgen.0.000362

**Published:** 2020-03-31

**Authors:** Amelia R. L. Charbonneau, Emma Taylor, Catriona J. Mitchell, Carl Robinson, Amy K. Cain, James A. Leigh, Duncan J. Maskell, Andrew S. Waller

**Affiliations:** ^1^​ Animal Health Trust, Lanwades Park, Newmarket, Suffolk, UK; ^2^​ Department of Veterinary Medicine, University of Cambridge, Cambridge, UK; ^3^​ School of Veterinary Medicine, University of Surrey, Guildford, UK; ^4^​ Department of Molecular Sciences, Macquarie University, Sydney, Australia; ^5^​ School of Veterinary Medicine and Science, University of Nottingham, Sutton Bonington, Leicestershire, UK; ^6^​ University of Melbourne, Victoria, Australia

**Keywords:** *Streptococcus equi*, transposon-directed insertion-site sequencing, whole blood, hydrogen peroxide

## Abstract

The availability of next-generation sequencing techniques provides an unprecedented opportunity for the assignment of gene function. *Streptococcus equi* subspecies *equi* is the causative agent of strangles in horses, one of the most prevalent and important diseases of equids worldwide. However, the live attenuated vaccines that are utilized to control this disease cause adverse reactions in some animals. Here, we employ transposon-directed insertion-site sequencing (TraDIS) to identify genes that are required for the fitness of *S. equi* in whole equine blood or in the presence of H_2_O_2_ to model selective pressures exerted by the equine immune response during infection. We report the fitness values of 1503 and 1471 genes, representing 94.5 and 92.5 % of non-essential genes in *S. equi *, following incubation in whole blood and in the presence of H_2_O_2_, respectively. Of these genes, 36 and 15 were identified as being important to the fitness of *S. equi* in whole blood or H_2_O_2_, respectively, with 14 genes being important in both conditions. Allelic replacement mutants were generated to validate the fitness results. Our data identify genes that are important for *S. equi* to resist aspects of the immune response *in vitro*, which can be exploited for the development of safer live attenuated vaccines to prevent strangles.

## Data Summary

The DNA sequences generated in this study have been deposited in the European Nucleotide Archive under the accession number PRJNA578912.

**Impact Statement**

Strangles, caused by *Streptococcus equi* subsp* . equi *, is one of the most frequently identified infectious diseases of horses worldwide, but the available live attenuated vaccines can survive for too long at the site of administration, leading to the development of adverse reactions. We applied a whole-genome approach to identify genes that are required in order for *S. equi* to grow in whole equine blood or in the presence of hydrogen peroxide, which simulate pressures exerted by the equine immune response. The simultaneous identification of every gene encoded within the DNA of *S. equi* that contributes to its ability to evade immune responses provides novel information about this important pathogen, and opens up exciting new opportunities for the design of safer and more effective vaccines with which to prevent strangles.

## Introduction

Strangles, caused by *Streptococcus equi* subspecies *equi *, is one of the most frequently diagnosed infectious diseases of equids worldwide, and is responsible for considerable economic and welfare cost to the horse industry [1]. Following entry via the nasopharyngeal or oral routes, *S. equi* subsp. *equi* binds to and invades the mucosal epithelium before transitioning to the lymph nodes of the head and neck, where it can be identified within 3 h [2]. The presence of *S. equi* subsp. *equi* within lymph nodes induces infiltration of polymorphonuclear leukocytes, leading to swelling and abscessation, which may cause dysphagia, lending to this disease's common name of strangles [3].

The earliest vaccines against strangles used heat-killed bacteria, but whilst conferring significant levels of protection, these vaccines led to severe adverse reactions [4–8]. Cell-free-extract vaccines have also been developed, but published data suggested that the protection conferred by such vaccines was short-lived at best and that adverse reactions at the injection site remained a problem [9]. Recombinant subunit vaccines benefit from enhanced safety, and a new multi-component vaccine, Strangvac 4, has been shown to confer significant levels of protection against *S. equi* subsp. *equi* at 2 weeks post-combined intranasal and subcutaneous vaccination [10, 11]. However, Strangvac 4 is not yet available for use in horses.

Two live attenuated vaccines are available commercially for the prevention of strangles: Pinnacle IN [12] and Equilis StrepE [13]. These vaccines confer protection against challenge with *S. equi* subsp. *equi *, but the attenuated vaccine strains can cause adverse reactions and even strangles in some vaccinated animals [14–17]. A prototype live attenuated vaccine containing deletions in six genes conferred high levels of protection at 2 months post-second vaccination, but also led to adverse reactions when administered via the intramuscular route [18]. *S. equi* subsp. *equi* resists the equine immune system by producing known factors such as streptolysin S, immunoglobulin cleaving enzymes, a factor H-binding protein, SeM, fibronectin-binding proteins and a protective hyaluronic acid capsule [18–26]. However, it is likely that several other factors, which remain unidentified, are also employed to resist the equine immune system. The identification and attenuation of such factors provides an opportunity to enhance the safety of live attenuated vaccines.

The increased accessibility of functional genomics techniques has facilitated the development of a variety of transposon-insertion sequencing methods, which combine dense random mutant libraries and next-generation sequencing to identify essential bacterial genomes and assign gene function [27–33]. Exposure of mutant libraries to varying experimental conditions enables the relative fitness and conditional essentiality of each gene to be determined either *in vitro* [27, 34–36] or *in vivo* [37–42].

We developed a novel barcoded transposon-directed insertion-site sequencing (TraDIS) technique in *S. equi* subsp. *equi* utilizing pGh9:IS*S1*, which produces random, dense and stable transposon libraries [32]. To identify any novel genes involved in the survival of *S. equi* subsp. *equi* in the face of the equine immune system, *S. equi* mutant libraries were exposed to two conditions: whole equine blood and Todd–Hewitt broth (THB) containing hydrogen peroxide (H_2_O_2_). To validate the findings, a panel of six allelic replacement mutants were exposed to whole equine blood and H_2_O_2_, and the impact on their viability was measured.

## Methods

### Bacterial strains, DNA isolation and primers

*S. equi* subsp. *equi* strain 4047 (*Se*4047) was grown in THB at 37 °C in a humidified atmosphere containing 5 % CO_2_, unless otherwise stated. The *Escherichia coli* strain TG1 *repA*+ was used for the replication of the pGhost9 IS*S1* plasmids at 37 °C. *S. equi* subsp. *equi* genomic DNA was extracted using GenElute spin columns (Sigma Aldrich), according to the manufacturer’s instructions. A list of all primers used in this study is available in Table S1 (available with the online version of this article).

### Minimum inhibitory concentration (MIC) of hydrogen peroxide (H_2_O_2_)

To determine the concentration of H_2_O_2_ required to exert a selective pressure on *S. equi* subsp* . equi *, the MIC of H_2_O_2_ in THB was determined. An overnight culture of *Se*4047 was diluted 40-fold and incubated until OD_600_ 0.3 was reached (containing approximately 2×10^8^ c.f.u. ml^−1^). In a conical bottom 0.2 ml 96-well plate, the culture was diluted such that each well contained 4×10^5^ c.f.u. ml^−1^ and doubling dilutions of THB containing H_2_O_2_, ranging from 1.5 to 0.00046 % (Sigma Aldrich). Wells containing ddH_2_O, instead of H_2_O_2_, were included as a control. The MIC was defined as the concentration of H_2_O_2_ in THB at which no growth of *Se*4047 occurred after incubation for 12 h at 37 °C in a humidified atmosphere containing 5 % CO_2_. The experiment was conducted in triplicate and repeated on three independent occasions.

### TraDIS in whole horse blood, H_2_O_2_ or THB

Three transposon libraries, AC, CT and GA described in our previous work [32], were each generated using a different modified IS*S1* transposon, such that the bases AC, CT or GA were located three bases downstream of the IS*S1* inverted repeat and adjacent to the genome sequence of *S. equi* subsp. *equi* within each transposon mutant. As each barcoded library was generated independently, each served as an experimental replicate. One millilitre of each stored *S. equi* subsp. *equi* barcoded transposon library was added to 39 ml of pre-warmed and pre-gassed THB containing 0.5 µg erythromycin ml^−1^ (THBE), resulting in cultures of approximately 0.05–0.08 OD_600_. Cultures were grown at 37 °C in a humidified atmosphere containing 5 % CO_2_ for approximately 3 h until OD_600_ 0.3 was reached. Five millilitres of the OD_600_ 0.3 cultures were centrifuged at 10000 rpm for 5 min, generating pellets representing the input population of mutants. The supernatants were removed, and the pellets frozen for DNA extraction. One hundred microlitres of each OD_600_ 0.3 culture was added to 50 ml of freshly drawn whole blood from the same Welsh mountain pony, THB containing 0.0004 % H_2_O_2_ (4×10^5^ c.f.u. ml^−1^) or THB (control). This number of bacteria was equivalent to approximately 66 c.f.u. and 72 c.f.u. of each mutant in the whole equine blood/THB control and H_2_O_2_ pools, respectively. The cultures were incubated for 2 h (whole blood and 0.0004 % H_2_O_2_) or overnight (THB control) at 37 °C in a humidified atmosphere containing 5 % CO_2_ with rotation (30 r.p.m.). An incubation time of 2 h in whole blood has been used previously to demonstrate the effects of deletion of the *hasA* gene [18]. To ensure output pools contained viable bacteria, mutants were recovered after incubation with whole blood or H_2_O_2_ by plating 300 µl onto 20 Todd–Hewitt agar (THA) Petri dishes containing 0.5 µg erythromycin ml^−1^ (THAE) and 0.03 µg hyaluronidase ml^−1^, followed by overnight incubation at 37 °C in a humidified atmosphere containing 5 % CO_2_. Hyaluronidase was included to facilitate the recovery of distinct colonies of surviving mutants. Mutant colonies were washed off the Petri dishes using THB containing 50 % glycerol (v/v) for direct storage at −20 °C. Two millilitres of the recovered mutants were centrifuged at 10 000 r.p.m. for 5 min, the supernatants removed and the pellets frozen for DNA extraction. Five millilitres of the overnight control cultures in THB were centrifuged at 10 000 r.p.m. for 5 min, the supernatants removed and the pellets frozen for DNA extraction. The input pool of mutants for the whole blood analysis also served as the input pool for comparison with the overnight THB control culture.

DNA was extracted from the input pellets of each of the three independent IS*S1* libraries and from each recovered library, which served as experimental replicates (three input and three output libraries). Purified DNA was sequenced by TraDIS, as previously described [32]. In brief, DNA was fragmented to approximately 600–800 bp, the fragment ends were repaired, and A-tailed and Y-adaptors ligated to the fragments. DNA was then digested with *Sma*I to minimize plasmid sequencing, and amplified by PCR with a specific IS*S1* primer and a unique indexing PCR primer for each of the six samples (indexing primers AHT 6, 7, 15, 16, 21 and 32 in Table S1). PCR products were purified, and size selected using AMPure XP beads. Libraries were quantified using the Kapa Biosystems library qPCR quantification kit and gel electrophoresis. Each prepared library was diluted to 2 nM and combined in equal concentrations to form a pool of the six uniquely indexed samples. PhiX (Illumina) was also diluted to 2 nM. The library pool and PhiX were denatured and neutralized, combined in the ratio of 3:2 and sequenced on an Illumina MiSeq DNA sequencer, as previously described [32].

The TraDIS sequencing data from the triplicate output and input samples were analysed as previously described using the BioTraDIS pipeline [32, 43] and the *Se*4047 reference genome [25]. Stringent mapping criteria of 100 % read match were imposed on the dataset resulting in between 37.8 and 57.6 % of reads mapping to the *Se*4047 reference genome (Table S2). Reads mapping to the final 10 % of each gene were discounted to prevent false negatives of gene fitness and function as the transposition of IS*S1* into this region of a gene can lead to no functional effect. Three genes (SEQ0285, SEQ0882 and SEQ1147) that were over-represented in the input pools due to the prevalence of a few specific IS*S1* mutants were also removed from the dataset. Read counts per gene were normalized between the input libraries to facilitate data comparison. Eighty-five genes that contained <10 reads mapping to them, in any one of the three normalized input libraries, were removed to ensure each gene was sufficiently represented to minimize the effects of stochastic loss. Five-hundred and seventy-five genes previously identified as essential, ambiguous or not defined for survival in THB were also removed from the analysis [32]. These criteria permitted the inclusion of 1503 genes in the whole blood and THB overnight control analyses, which represent 94.5 % of non-essential genes in *S. equi* subsp* . equi *. A total of 1471 genes met the same inclusion criteria in the H_2_O_2_ analysis, which represented 92.5 % of non-essential genes in *S. equi* subsp. *equi* [32]. The three input pools contained on average 17 and 16 different IS*S1* mutants in each of the 1503 and 1471 genes that passed filtering, respectively. All genes removed from the input data were similarly removed from the output data before the read counts per gene were normalized between the output libraries. The script tradis_comparison was then used to compare each of the three sets of three output libraries to the three input libraries, on a sequencing reads per gene basis, generating a fitness value [log_2_ fold change (FC)], *P* and *q* value for each gene. Genes were considered to have decreased fitness if they exhibited a log_2_FC value of less than −2 compared to the input control and had a *q* value of<0.05.

### Validation of TraDIS whole equine blood and H_2_O_2_ fitness results

To confirm the reduced fitness of some genes as reported by TraDIS, allelic replacement mutants in *Se*4047 were generated lacking the genes *pyrP* (SEQ1316), *mnmE* (SEQ1365), *addA* (SEQ0953) and *recG* (SEQ0454). Strains of *Se*4047 lacking *hasA* (SEQ0269) and *eqbE* (SEQ1242) were also utilized, both of which have been described previously [18, 44]. The Δ*hasA* strain was used as a positive control in the whole equine blood assay, as it is known to be attenuated under this condition [18]. The Δ*eqbE* strain was used as a negative control, as TraDIS data showed that fitness in whole blood was not altered upon insertion of IS*S1*.

Deletion mutants were generated using an allelic replacement mutagenesis technique, as previously described for the generation of a Δ*prtM* mutant [45]. Briefly, 500 bp regions of *S. equi* subsp. *equi* DNA flanking the target gene were amplified using the primers listed in Table S1, ligated together and cloned into the pGhost9 plasmid [45]. Constructs were used individually to transform competent *Se*4047 cells, which were grown on THAE at 28 °C (the plasmid permissive temperature) for 2 days. Single colonies were inoculated into THBE overnight at 28 °C and then transferred to 37 °C for 3 h to induce chromosomal integration of the construct. Integrants were selected on THAE overnight at 37 °C; then, they were grown overnight at 37 °C in THBE, followed by dilution into THB and incubation at 28 °C for 48 h to excise pGhost9 and the target gene from the chromosome. Excised bacteria were grown on THA overnight at 37 °C to ensure free plasmid was lost. To confirm plasmid loss and, therefore, loss of erythromycin resistance, deletion strains were grown on both THAE and THA. Mutant alleles were confirmed by PCR using the appropriate P1 and P4 primers (suffixed with the gene name in Table S1) and sequencing on an ABI3100 DNA sequencer using BigDye fluorescent terminators. Deletion strains were stored in 25 % glycerol (v/v) at −80 °C.

### Growth curves of validation strains

A single colony of each deletion mutant and *Se*4047 were inoculated into 10 ml THB in triplicate and grown for 16 h. Cultures were then diluted to approximately OD_600_ 0.08 in pre-warmed and pre-gassed THB, and grown until stationary phase was reached. The mean OD_600_ across the three replicates of each strain and their se values were calculated. The doubling time of each replicate of each strain was calculated from exponential phase data and used to identify significant differences in growth rates of mutants compared to wild-type *Se*4047 using a two-tailed Student’s *t*-test.

### Whole equine blood and H_2_O_2_ validation assays

Three overnight cultures for each deletion strain and six overnight cultures of *Se*4047 were generated by inoculating 10 ml THB with a single colony. Cultures were grown for 16 h, then diluted to OD_600_ 0.08 in pre-warmed and pre-gassed THB. Cultures were grown to OD_600_ 0.3 and then diluted to 1×10^5^ c.f.u. ml^−1^ in THB. One hundred microlitres was then added to 10 ml freshly drawn blood from the same Welsh mountain pony that was used in the TraDIS study or THB containing 0.0004 % H_2_O_2_ and incubated for 2 h with rotation (30 r.p.m.). Immediately after adding the strains, 50 µl was plated neat, in triplicate, onto Columbia CNA staph/strep selective agar (Oxoid) [time point 0 (T0)] to enumerate the initial concentration of *S. equi* subsp. *equi* cells. Surviving cells were enumerated at 1 (T1), 2 (T2) and 3 h (T3) by plating various dilutions in PBS onto CNA agar in triplicate. Colony counts for each set of triplicate Petri dishes were converted into a mean c.f.u. ml^−1^ for each time point for each replicate. Mean c.f.u. ml^−1^ data from T1, T2 and T3 were transformed into a percentage relative to T0 within each replicate to normalize the data, as the T0 c.f.u. ml^−1^ varied slightly between experiments. The three values of transformed data per deletion strain at each time point were used to calculate the doubling time of each replicate of each strain, which was compared to wild-type *Se*4047 using a two-tailed Student’s *t*-test.

## Results

### Identification of genes important for fitness in whole equine blood

The three barcoded IS*S1* libraries designated AC, CT and GA, described in our previous work [32], were grown to an OD_600_ of 0.3 immediately before use and re-sequenced to accurately identify input pool composition. Post-filtering, input libraries contained 26 381 unique mutants in library AC, 24 353 unique mutants in library CT and 28 128 in library GA (Table S3), representing 1503 (94.5 %) of the 1590 non-essential genes previously identified in *S. equi *.

**Fig. 1.**
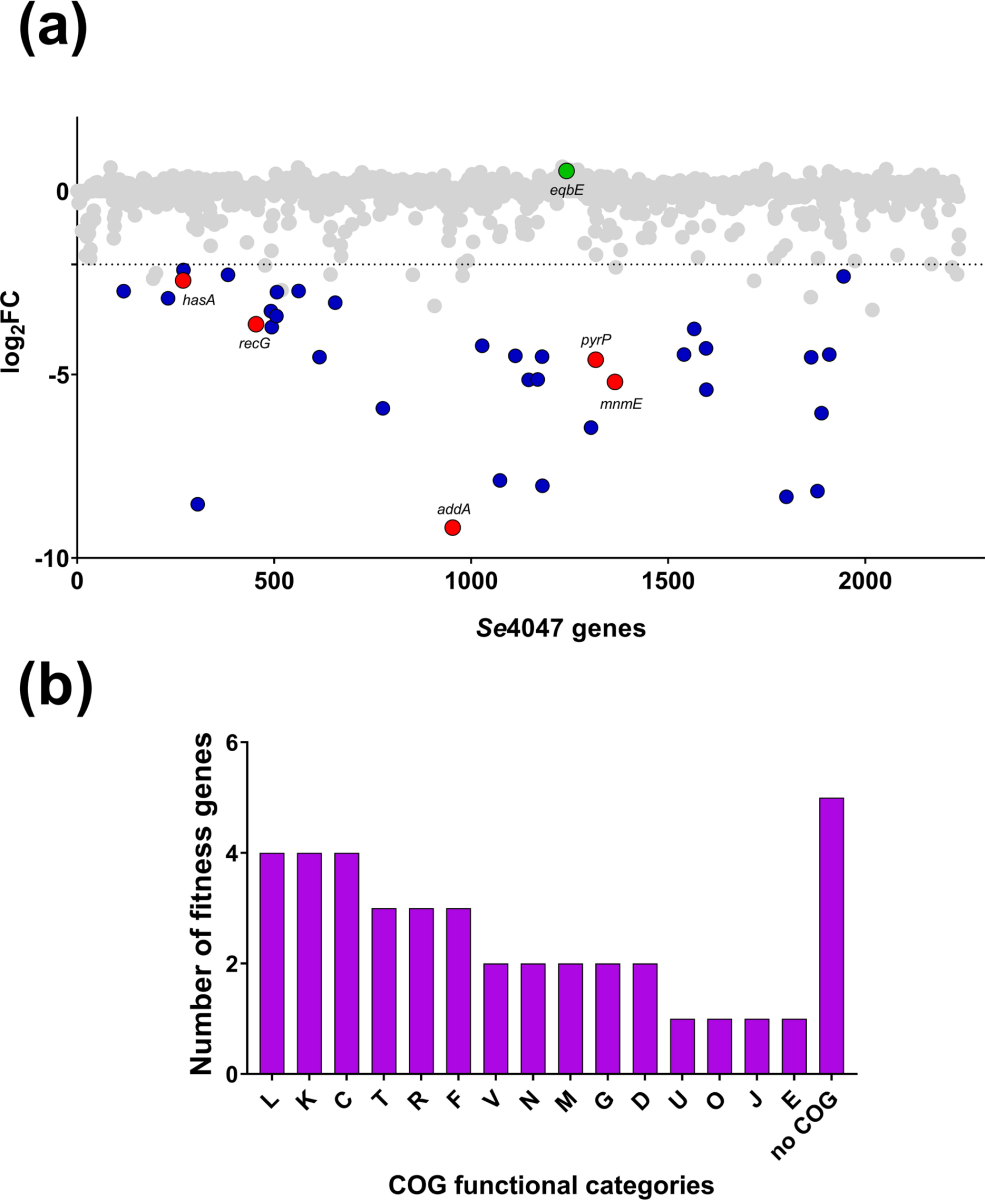
Fitness scores and COG categories of *S. equi* subsp. *equi* genes required for survival in whole equine blood. (a) Fitness scores (log_2_FC) per gene of *S. equi* subsp. *equi* IS*S1* mutants post-incubation in whole equine blood, as determined by TraDIS. Blue dots, genes with significantly reduced fitness (log_2_FC < −2 and *q*<0.05); red dots, genes significantly reduced in fitness of which deletion mutants were made and retested to confirm TraDIS results; green dot, *eqbE* exhibiting no fitness effect that was used as a control for validation; grey dots, genes exhibiting no significant fitness effect. (b) Functional COG categories of the 36 fitness genes identified in whole equine blood. L, Replication, recombination and repair; K, transcription; C, energy production and conversion; T, signal transduction mechanisms; R, general function prediction only; F, nucleotide transport and metabolism; V, defence mechanisms; N, cell motility; M, cell wall/membrane/envelope biogenesis; G, carbohydrate transport and metabolism; D, cell cycle control, cell division, chromosome partitioning; U, intracellular trafficking, secretion and vesicular transport; O, posttranslational modification, protein turnover, chaperones; J, translation, ribosomal structure and biogenesis; E, amino acid transport and metabolism.

The barcoded mutant libraries were each exposed to whole equine blood and the genes contributing to fitness in this environment were identified by TraDIS. The three barcoded libraries recovered from whole equine blood contained, on average, 9.4±3.5 % (sem) fewer unique mutants than were present in the input libraries (Table S3). The log_2_FC was calculated for all 1503 genes included in the analysis (Fig. 1a). Thirty-six genes were significantly reduced in fitness in whole equine blood (Fig. 1a, blue and red dots, Tables 1 and S4). The remaining 1466 genes exhibited no growth defects in whole equine blood as a result of IS*S1* insertion (Fig. 1a, grey dots and green dot). Cluster of orthologous groups (COG) analysis of the 36 fitness genes (Fig. 1b) identified that the most prevalent categories included genes involved in replication, recombination and repair (*n*=4), transcription (*n*=4), and energy production and conversion (*n*=4). Five genes (14 % of fitness genes) did not belong to a defined COG category.

**Table 1.** *
S. equi* subsp. *equi* genes with reduced fitness in equine whole blood as a result of IS*S1* insertion as identified by TraDIS. Genes highlighted in grey were deleted by allelic replacement mutagenesis and deletion strains incubated in whole equine blood to validate TraDIS results. An Δ*eqbE* deletion strain was used as a negative control

Gene	Locus tag	Function	log_2_FC	*q* value
*ackA*	SEQ0118	Acetate kinase	−2.7	0.042
*SEQ0231*	SEQ0231	Putative Mga-like regulatory protein	−2.9	<0.0005
*hasA*	SEQ0269	Hyaluronan synthase	−2.4	0.046
*hasB*	SEQ0270	UDP-glucose 6-dehydrogenase	−2.2	<0.0005
*SEQ0306*	SEQ0306	Putative ssDNA-binding protein	−8.5	<0.0005
*pepX*	SEQ0383	Xaa-Pro dipeptidyl-peptidase	−2.3	0.017
*recG*	SEQ0454	ATP-dependent DNA helicase	−3.6	0.001
*SEQ0492*	SEQ0492	Putative mannose-specific phosphotransferase system (PTS), IID component	−3.3	0.042
*SEQ0494*	SEQ0494	Putative mannose-specific phosphotransferase system (PTS), IIAB component	−3.7	0.017
*pptA/ecsA*	SEQ0506	ABC transporter ATP-binding protein	−3.4	0.021
*pptB/ecsB*	SEQ0507	ABC transporter protein	−2.8	0.002
*SEQ0562*	SEQ0562	Exodeoxyribonuclease	−2.7	0.022
*bipA/typA*	SEQ0615	GTPase	−4.5	0.007
*pyrD*	SEQ0655	Putative dihydroorotate dehydrogenase	−3.0	0.007
*ppc*	SEQ0776	Putative phosphoenolpyruvate carboxylase	−5.9	<0.0005
*addA*	SEQ0953	Putative ATP-dependent exonuclease subunit A	−9.2	<0.0005
*SEQ1028*	SEQ1028	GntR family regulatory protein	−4.2	0.004
*SEQ1073*	SEQ1073	Putative phosphopantothenoylcysteine decarboxylase	−7.9	<0.0005
*SEQ1112*	SEQ1112	Putative exported protein	−4.5	0.001
*SEQ1146*	SEQ1146	Putative phosphate acetyltransferase	−5.1	<0.0005
*ldh*	SEQ1169	l-Lactate dehydrogenase	−5.1	<0.0005
*SEQ1180*	SEQ1180	Putative DNA-binding protein	−4.5	0.003
*SEQ1181*	SEQ1181	GntR family regulatory protein	−8.0	<0.0005
*SEQ1304*	SEQ1304	Pyridine nucleotide-disulphide oxidoreductase family protein	−6.4	<0.0005
*pyrP*	SEQ1316	Uracil permease	−4.6	<0.0005
*mnmE*	SEQ1365	tRNA modification GTPase	−5.2	<0.0005
*SEQ1540*	SEQ1540	Putative membrane protein	−4.5	0.003
*smc*	SEQ1566	Putative chromosome partition protein	−3.8	<0.0005
*ccpA*	SEQ1596	Catabolite control protein A	−4.3	0.011
*pepQ*	SEQ1597	Putative Xaa-Pro dipeptidase	−5.4	<0.0005
*SEQ1800*	SEQ1800	Putative exported protein	−8.3	<0.0005
*scpA*	SEQ1863	Segregation and condensation protein A	−4.5	<0.0005
*greA*	SEQ1879	Transcription elongation factor	−8.2	<0.0005
*csrS*	SEQ1889	Sensor histidine kinase	−6.1	<0.0005
*yqeK*	SEQ1909	Hydrolase, HD family	−4.5	0.002
*pyrG*	SEQ1945	Putative CTP synthase	−2.3	<0.0005
*eqbE*	SEQ1242	Equibactin nonribosomal peptide synthase protein	0.6	1

### Identification of genes important for fitness in hydrogen peroxide

**Fig. 2.**
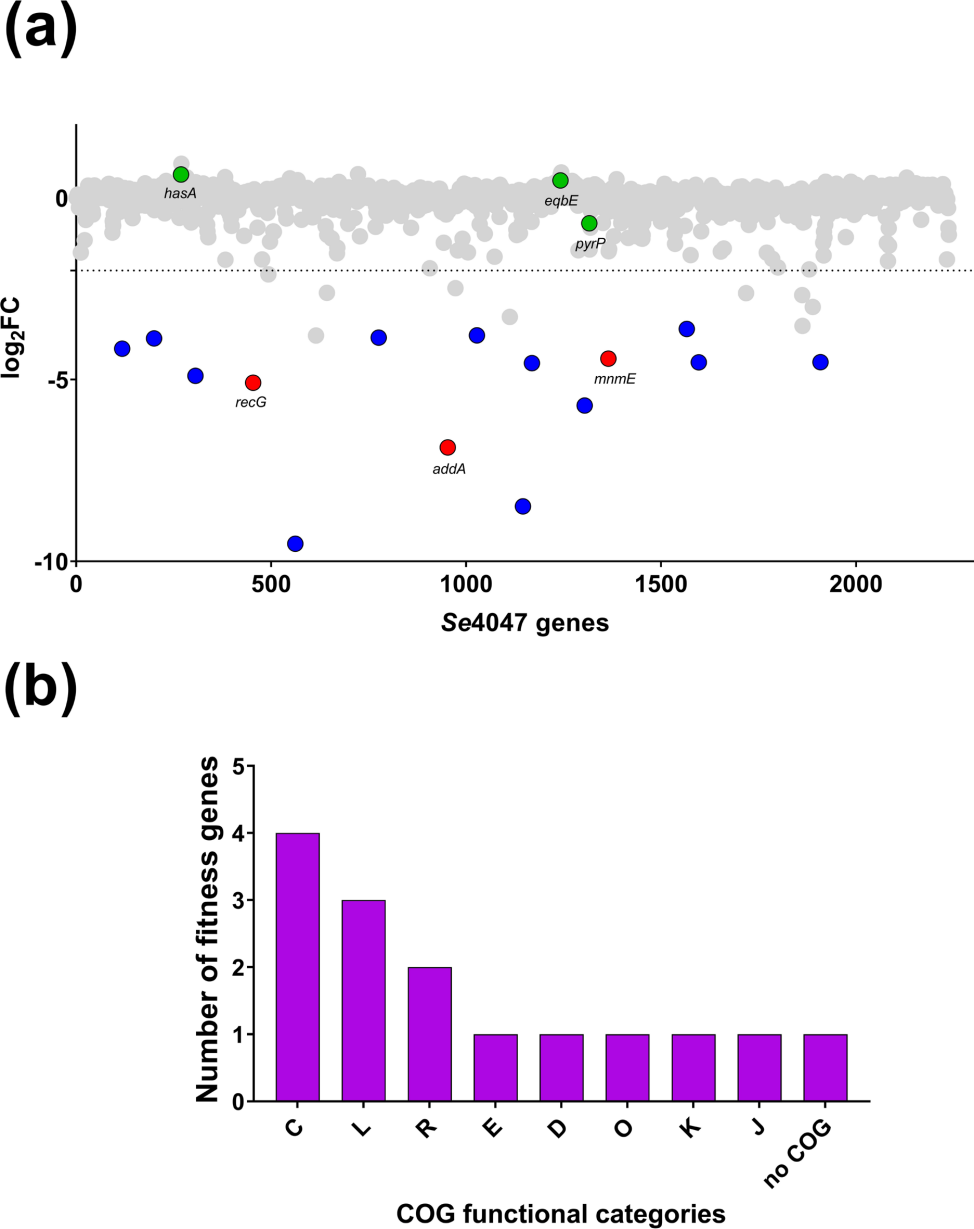
Fitness scores and COG categories of *S. equi* subsp. *equi* genes required for survival in hydrogen peroxide (H_2_O_2_). (a) Fitness scores (log_2_FC) per gene of *S. equi* subsp. *equi* IS*S1* mutants post-incubation in H_2_O_2_, as determined by TraDIS. Blue dots, genes with significantly reduced fitness (log_2_FC < −2 and q<0.05); red dots, genes significantly reduced in fitness of which deletion mutants were made and retested to confirm TraDIS results; green dots, genes exhibiting no fitness effect that acted as negative controls for validation; grey dots, genes exhibiting no significant fitness effect. (b) Functional COG categories of the fitness genes identified in H_2_O_2_. C, Energy production and conversion; L, replication, recombination and repair; R, general function prediction only; E, amino acid transport and metabolism; D, cell cycle control, cell division, chromosome partitioning; O, posttranslational modification, protein turnover, chaperones; K, transcription; J, translation, ribosomal structure and biogenesis.

**Fig. 3.**
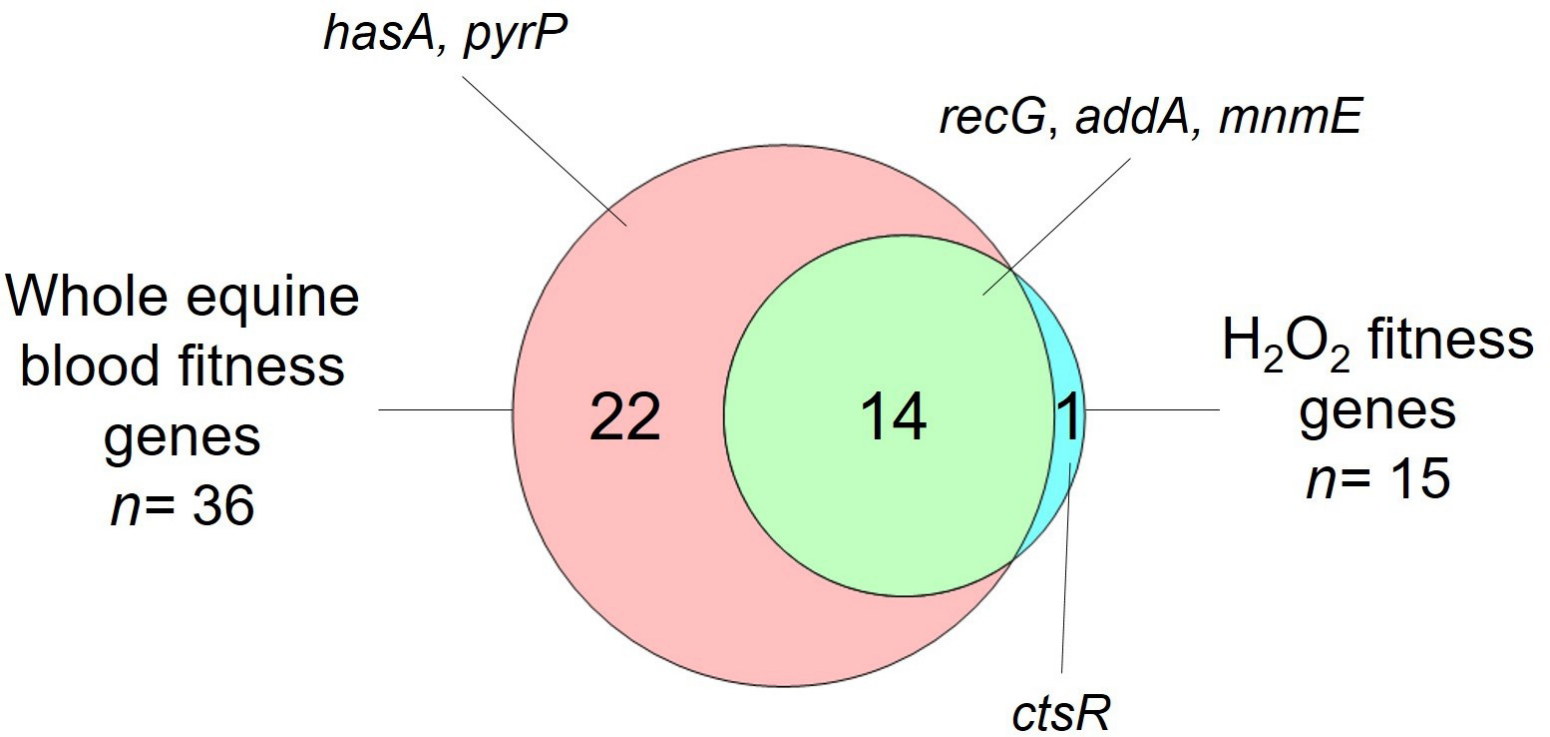
Venn diagram of the 36 genes required for the survival of *S. equi* subsp. *equi* in whole equine blood compared to the 15 genes required for survival in hydrogen peroxide. The genes that were deleted by allelic replacement mutagenesis to validate the results are indicated.

After filtering, input libraries contained 24 372 unique mutants in library AC, 22 734 unique mutants in library CT and 26 226 unique mutants in library GA (Table S5), which represented 92.5 % of the non-essential genes in *S. equi* subsp. *equi* [32]. The three output libraries recovered after H_2_O_2_ treatment contained, on average, 2.1±6.3 % (sem) fewer unique mutants than were present in the input libraries (Table S5). The effect of incubation with H_2_O_2_ on the fitness of IS*S1* mutants was determined by calculating the log_2_FC for all 1471 genes passing the inclusion criteria (Fig. 2a). Fifteen genes were significantly reduced in fitness (log_2_FC < −2 and *q*<0.05; Fig. 2a, blue and red dots, Tables 2 and S6), with the remaining 1456 genes exhibiting no growth defect in the presence of H_2_O_2_ as a result of IS*S1* insertion (Fig. 2a, grey and green dots). COG analysis of the 15 fitness genes (Fig. 2b) identified that the most prevalent categories included genes involved in energy production and conversion (*n*=4), and replication, recombination and repair (*n*=3). Fourteen of the fifteen genes identified in the H_2_O_2_ TraDIS screen were also implicated in survival in whole equine blood (Table 2, Fig. 3). One gene, *ctsR*, was uniquely identified in the H_2_O_2_ TraDIS screen (Table 2, highlighted in blue).

**Table 2.** *
S. equi* subsp. *equi* genes with reduced fitness in the presence of hydrogen peroxide (H_2_O_2_) as a result of IS*S1* insertion, as identified by TraDIS. One gene highlighted in blue was uniquely identified in the presence of H_2_O_2_ when compared to genes identified as reduced in fitness in whole equine blood. The remaining genes were similarly identified as required in whole equine blood. The genes highlighted in grey were deleted by allelic replacement mutagenesis and deletion strains incubated in THB containing H_2_O_2_ to validate TraDIS results. The Δ*eqbE* deletion strain was used as a control.

Gene	Locus tag	Function	log_2_FC	*q* value
*SEQ0118*	SEQ0118	Acetate kinase	−4.1	0.0021
*ctsR*	SEQ0200	Transcriptional regulator	−3.9	0.0221
*SEQ0306*	SEQ0306	Putative ssDNA-binding protein	−4.9	<0.0005
*recG*	SEQ0454	ATP-dependent DNA helicase	−5.1	<0.0005
*SEQ0562*	SEQ0562	Exodeoxyribonuclease	−9.5	<0.0005
*ppc*	SEQ0776	Putative phosphoenolpyruvate carboxylase	−3.8	0.0221
*addA*	SEQ0953	Putative ATP-dependent exonuclease subunit A	−6.9	<0.0005
*SEQ1028*	SEQ1028	GntR family regulatory protein	−3.8	0.0071
*SEQ1146*	SEQ1146	Putative phosphate acetyltransferase	−8.5	<0.0005
*ldh*	SEQ1169	l-Lactate dehydrogenase	−4.5	0.0015
*SEQ1304*	SEQ1304	Pyridine nucleotide-disulphide oxidoreductase family protein	−5.7	<0.0005
*mnmE*	SEQ1365	tRNA modification GTPase	−4.4	<0.0005
*smc*	SEQ1566	Putative chromosome partition protein	−3.6	<0.0005
*pepQ*	SEQ1597	Putative Xaa-Pro dipeptidase	−4.5	<0.0005
*yqeK*	SEQ1909	Hydrolase, HD family	−4.5	0.0009
*hasA*	SEQ0269	Hyaluronan synthase	0.6	1
*pyrP*	SEQ1316	Uracil permease	−0.7	1
*eqbE*	SEQ1242	Equibactin nonribosomal peptide synthase protein	0.5	1

### Generation of allelic replacement mutants in putative fitness genes

To validate the findings of the TraDIS fitness screen, we generated four allelic replacement mutants lacking the genes *recG, addA*, *pyrP* and *mnmE* to confirm single mutant fitness effects in selective conditions. *addA* was selected for validation as it was the gene most negatively affected in whole equine blood as a result of IS*S1* insertion. *recG* was selected because it was one of the least affected of the genes required for fitness in whole equine blood; in addition, a *recA* deletion has been incorporated previously into a live attenuated vaccine strain [18]. *pyrP* and *mnmE* were selected as these represented a middle ground in the affected genes. The Δ*hasA* and Δ*eqbE* mutants were generated previously. The Δ*hasA* mutant was utilized as it is known that this mutant has reduced fitness in whole equine blood [18], whilst the Δ*eqbE* allelic replacement mutant [44] was utilized as a negative control as IS*S1* mutants in this gene exhibited no attenuation in whole equine blood or H_2_O_2_.

**Fig. 4.**
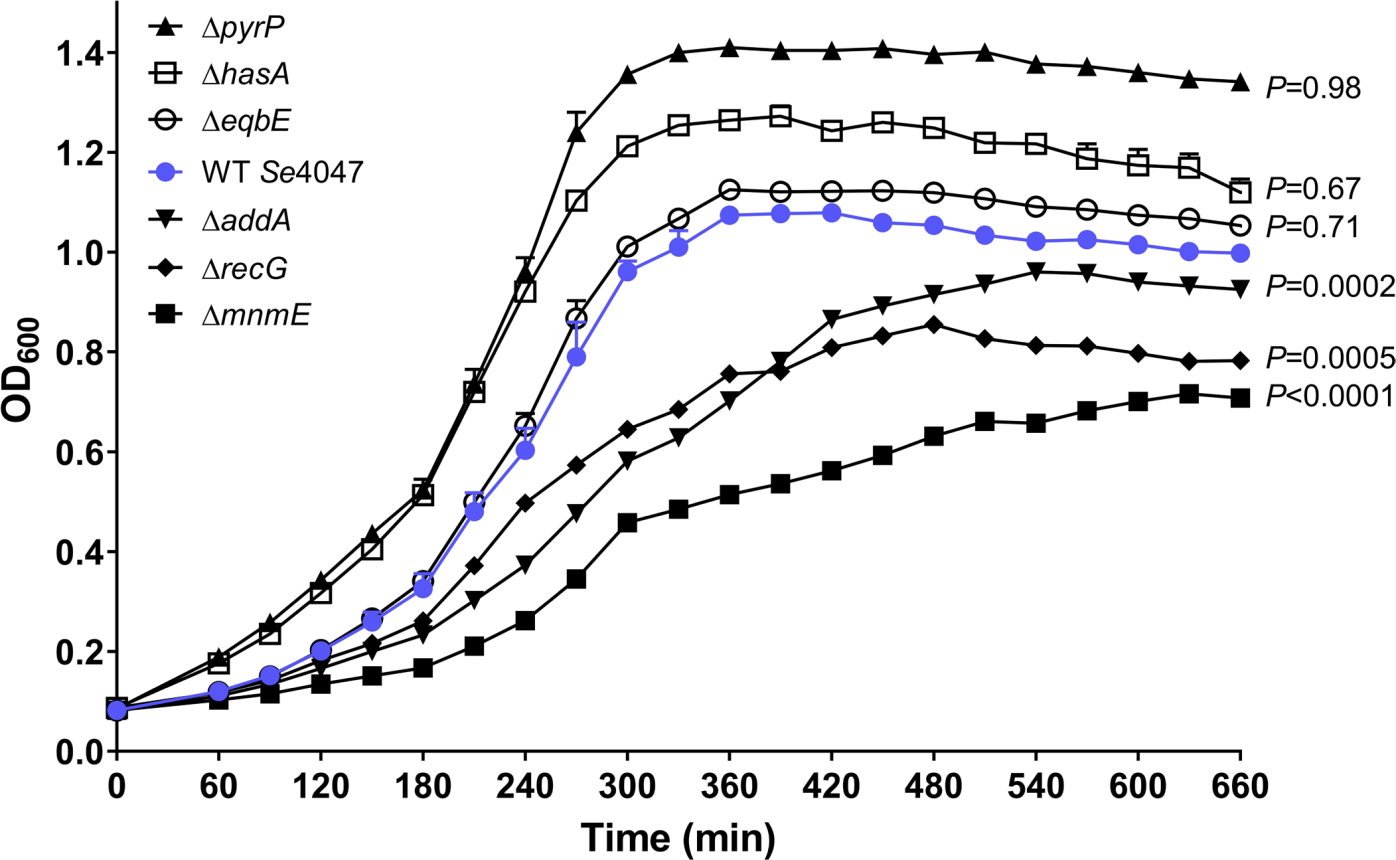
Growth curves of the parental *S. equi* subsp. *equi* strain *Se*4047 and Δ*pyrP*, Δ*hasA,* Δ*eqbE,* Δ*addA*, Δ*recG* and Δ*mnmE* deletion mutation strains grown in THB at 37 °C in a humidified atmosphere in the presence of 5 % CO_2_. Error bars indicate the se. The significance of changes in doubling time using a two-sided Student’s *t*-test are indicated.

Deletion strains were grown in THB and optical densities measured over time to determine their growth characteristics (Fig. 4). The Δ*addA* (*P*=0.0002), Δ*recG* (*P*=0.0005) and Δ*mnmE* (*P*<0.0001) deletion strains grew significantly more slowly than *Se*4047 in THB, despite these genes being identified previously as non-essential [32].

To determine whether the identification of genes that were important for the fitness of *S. equi* subsp. *equi* in the presence of whole equine blood or H_2_O_2_ by TraDIS was due to a generalized fitness defect from the increased time that mutation strains were grown in THB during these experiments, we compared the mutant populations of the three barcoded libraries recovered post-overnight incubation in THB with the input mutant pool immediately prior to growth in whole equine blood. Six genes (*pyrD*, *sufB*, *sufD*, SEQ1930, SEQ2142 and SEQ2146) were identified as having significantly reduced fitness in THB (Table S7). One of these genes, *pyrD* had indeed been identified as having reduced fitness in whole equine blood.

### Survival of key mutation strains in whole blood

**Fig. 5.**
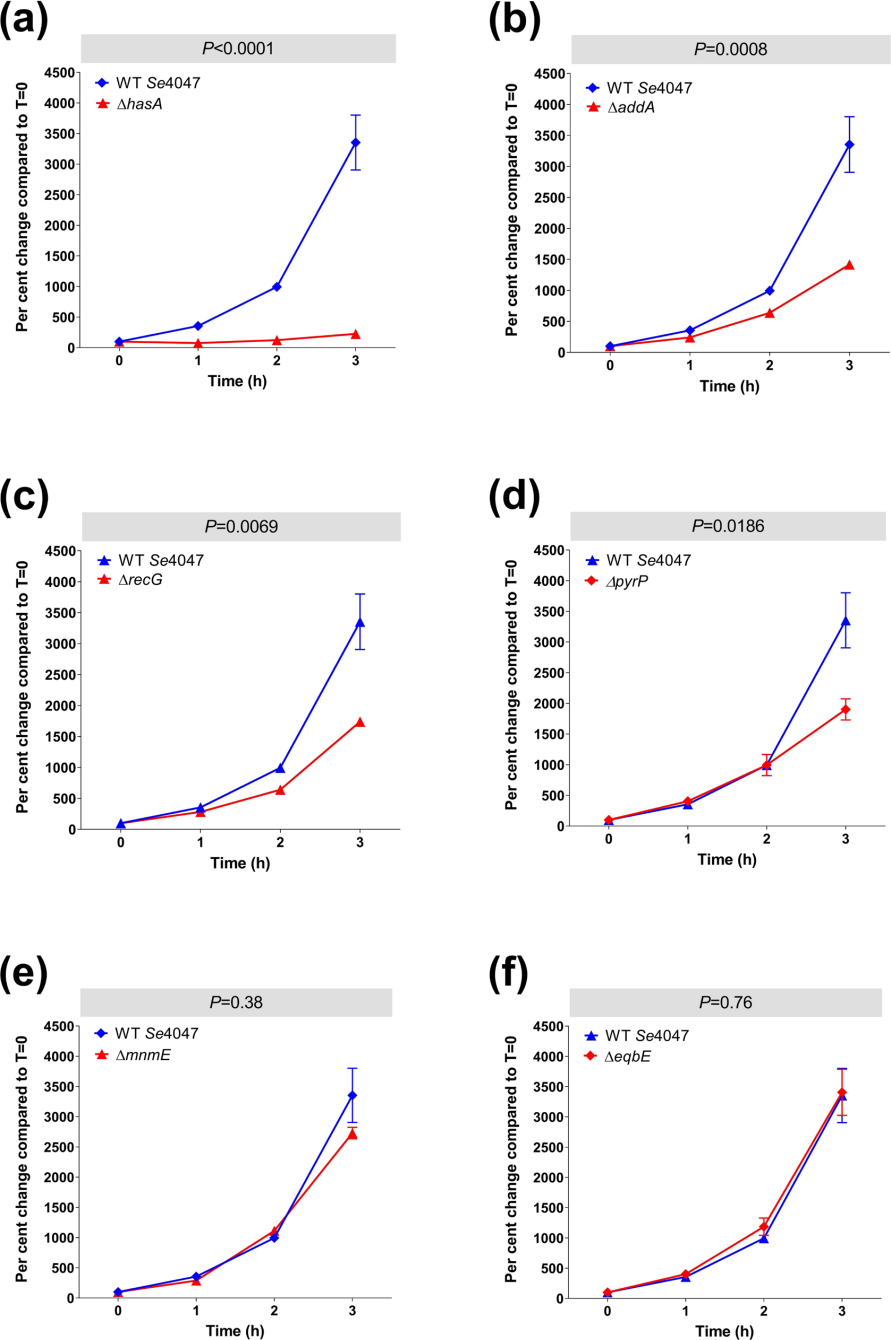
Validation of the *S. equi* subsp. *equi* TraDIS screen in whole equine blood. Strains with deletion mutations of whole equine blood fitness genes, as identified by TraDIS, were incubated in blood for 3 h and their survival measured each hour. (a) Δ*hasA*, (b) Δ*addA*, (c) Δ*recG*, (d) Δ*pyrP*, (e) Δ*mnmE* and (f) Δ*eqbE* deletion mutation strains compared to the wild-type parental strain, *Se*4047. Error bars indicate the se. The significance of changes in doubling time using a two-sided Student’s *t*-test are indicated.

The six deletion strains were incubated in whole equine blood with reduced bacterial loads to more closely reflect the proportion of attenuated *S. equi* subsp. *equi* IS*S1* mutants present in the original TraDIS assay. Validation assays were also incubated for an additional hour. The growth of each deletion strain in whole equine blood was measured over time and compared statistically to wild-type *Se*4047 (Fig. 5). The Δ*hasA* (*P*<0.0001) strain was highly attenuated in whole equine blood with a significantly longer doubling time (Fig. 5a), which is in agreement with published data on this strain [18]. The doubling times of the Δ*addA* (*P*=0.0008), Δ*recG* (*P*=0.0069) and Δ*pyrP* (*P*=0.019) strains were significantly longer than wild-type *Se*4047 in whole equine blood (Fig. 5b–d). However, the Δ*mnmE* strain (*P*=0.38) did not have a significantly longer doubling time than *Se*4047 in whole equine blood (Fig. 5e). The Δ*eqbE* control strain grew at the same rate as *Se*4047 (Fig. 5f).

### Survival of mutation strains in H_2_O_2_

**Fig. 6.**
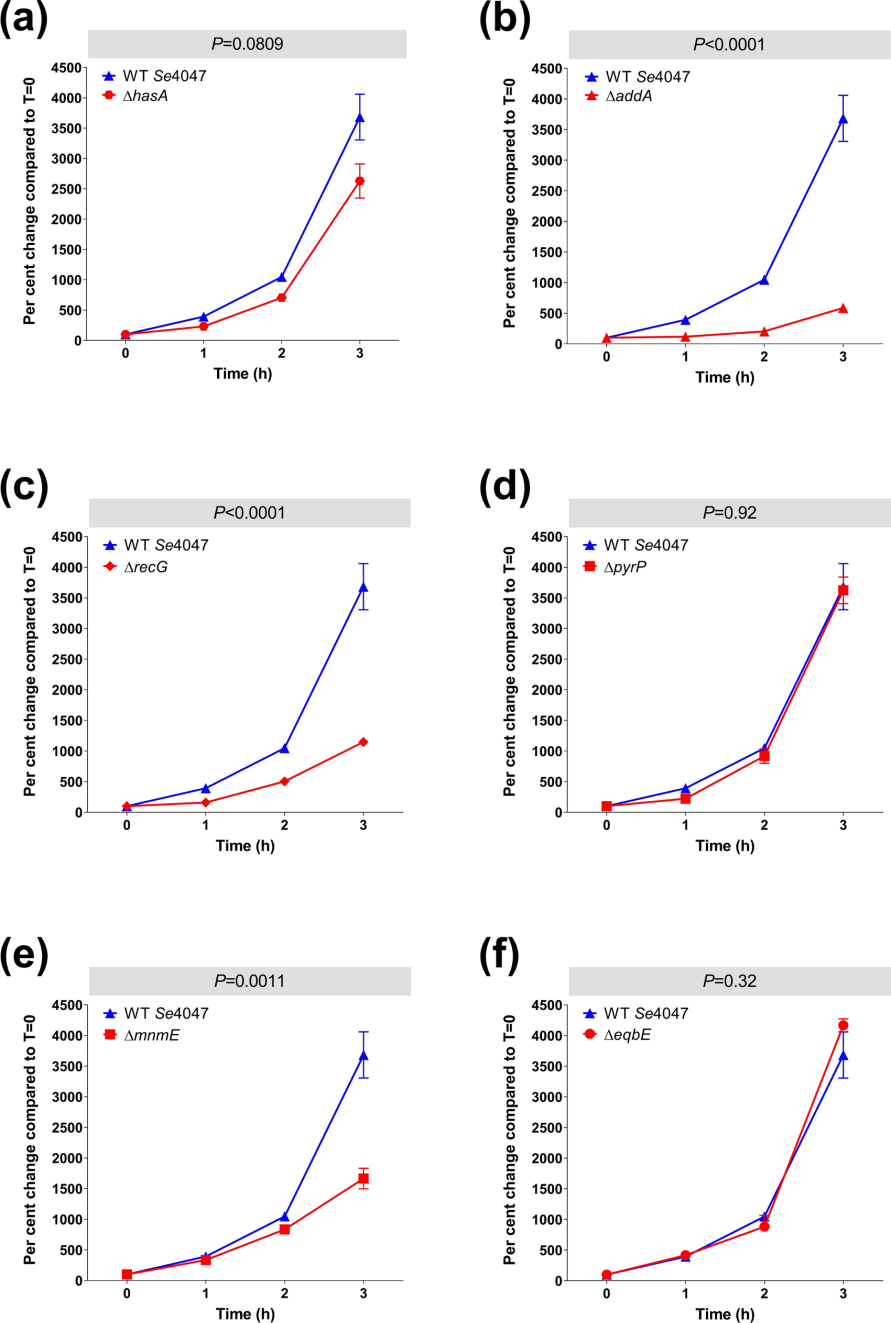
Validation of the *S. equi* subsp. *equi* TraDIS screen in THB containing hydrogen peroxide. Strains with deletion mutations in H_2_O_2_ fitness genes, as identified by TraDIS, were incubated in THB containing H_2_O_2_ for 3 h and their survival measured each hour. (a) Δ*hasA*, (b) Δ*addA*, (c) Δ*recG*, (d) Δ*pyrP*, (e) Δ*mnmE* and (f) Δ*eqbE* deletion mutation strains compared to the wild-type parental strain, *Se*4047. Error bars indicate the se. The significance of changes in doubling time using a two-sided Student’s *t*-test are indicated.

The growth of each allelic replacement mutation strain in the presence of sub-MIC concentrations of H_2_O_2_ was measured over time and compared statistically to the growth of *Se*4047 (Fig. 6). The doubling times for the Δ*addA* (*P*<0.0001), Δ*recG* (*P*<0.0001) and Δ*mnmE* (*P*=0.0011) strains in the presence of H_2_O_2_ were significantly longer than that of *Se*4047 (Fig. 6b, c and e). The insertion of IS*S1* into *hasA*, *pyrP* or *eqbE* did not confer any fitness defects in H_2_O_2_ (Table 2). In agreement with these data, the Δ*hasA*, Δ*pyrP* and Δ*eqbE* mutants grew at similar rates to *Se*4047 in the presence of H_2_O_2_(Fig. 6a, d and f).

## Discussion

Here, we describe the genome-wide identification of genes required by *S. equi* subsp. *equi* for survival in the presence of whole equine blood and H_2_O_2_, conditions that mimic an interaction with the equine immune response. IS*S1* mutants in 36 and 15 genes were significantly reduced in fitness upon exposure to whole equine blood or H_2_O_2_, respectively. Fourteen genes were required for fitness in both of these conditions. Four novel allelic replacement mutants lacking *addA, recG*, *pyrP* or *mnmE* were generated and tested to determine whether TraDIS had indeed identified novel genes that contribute to fitness in the presence of whole blood or H_2_O_2_. Two control mutants were also tested, a capsule deletion mutant, Δ*hasA* [18], and a Δ*eqbE* mutant [44].

*addAB* (also known as *rexAB*) encodes a major component of the homologous recombination process that repairs double-strand breaks by catalysing the unwinding of DNA [46–48]. In *S. equi *, *addB* was essential for growth in THB (insertion index=0.03, essential genes<0.034 [32]). Although, *addA* was not essential for growth *in vitro* or for fitness on overnight culture of the mutant libraries in THB, the Δ*addA* mutant grew more slowly than the wild-type strain (Fig. 4). This slow growth phenotype was also observed in *Streptococcus pneumoniae* Δ*addA* and Δ*addB* mutants [47]. The *S. equi* subsp. *equi* Δ*addA* deletion mutant was confirmed to be significantly attenuated in whole equine blood and H_2_O_2_. However, further investigation of the role of AddA is required as this result could, potentially, be related to the slow-growth phenotype of mutants lacking production of AddA.

*recG* encodes an ATP-dependent DNA helicase that is thought to be important for efficient recombination and DNA repair. RecG promotes the resolution of Holliday junctions by catalysing the conversion of junction intermediates to mature products by branch migration [49]. RecG is also thought to remove RNA from R-loops by unwinding the RNA–DNA hybrid [50, 51]. Although *recG* was non-essential for growth *in vitro* or for fitness on overnight culture of the mutant libraries in THB, the Δ*recG* mutant was found to have a slower growth rate (Fig. 4). *recG* IS*S1* mutants were more significantly attenuated in H_2_O_2_ compared to whole equine blood and this result was confirmed using the Δ*recG* mutant (Figs 5 and 6). This result could, in part, be related to the slow-growth phenotype of mutants lacking production of RecG.

A membrane-bound uracil permease encoded by *pyrP* scavenges uracil from the environment for pyrimidine biosynthesis [52]. *pyrP* was required for fitness in whole equine blood, but not in H_2_O_2_ (Tables 1 and 2). The Δ*pyrP* deletion mutant had a similar growth rate to the wild-type *Se*4047 strain in THB. In agreement with the TraDIS data, the Δ*pyrP* strain had a significantly longer doubling time in whole equine blood, but a similar doubling time to *Se*4047 in the presence of H_2_O_2_. Interestingly, *pyrD* and *pyrG*, which are involved in the downstream biosynthetic pyrimidine pathway, were also required by *S. equi* subsp. *equi* for fitness in the presence of whole equine blood, but not H_2_O_2_ *in vitro*. Although IS*S1* mutants in *pyrD* had significantly reduced fitness following overnight culture in THB (Table S7).

*mnmE* (also known as *trmE*) is predicted to encode a tRNA modification enzyme that forms a heterotetrameric complex with MnmG (also known as GidA) [53, 54]. The MnmEG complex catalyses two different GTP- and FAD-dependent reactions, generating 5-aminomethyluridine and 5-carboxymethylaminomethyluridine, utilizing ammonium and glycine as substrates, respectively [53]. GTP hydrolysis by MnmE causes structural rearrangements within the MnmEG complex, which is necessary for subsequent tRNA modification, in *E. coli* [55]. In *S. equi *, MnmG was found to be essential for growth *in vitro* [32], and it was critical for survival in *Streptococcus pyogenes* and *Streptococcus agalactiae* [32, 56, 57]. *S. pyogenes* Δ*mnmE* and Δ*mnmG* deletion mutants had decreased production of known virulence factors including streptolysin O, M-protein, mitogenic factor and NAD-glycohydrolase [58]. Deletion of *mnmE* or *mnmG* also decreased biofilm production by 50 % in *Streptococcus mutans* [59]. The growth rate of the *S. equi* subsp. *equi* Δ*mnmE* deletion mutation strain in THB was significantly decreased relative to *Se*4047 and Δ*mnmE* was the slowest growing of the allelic replacement mutants generated in this study (Fig. 4). IS*S1* mutants of *mnmE* had reduced fitness in both whole equine blood and H_2_O_2_. However, the Δ*mnmE* deletion mutation strain was only found to have a significantly longer doubling time in the presence of H_2_O_2_.

Streptococcal capsule mutants have long been known to be susceptible to killing in both *in vitro* and *in vivo* [60–63]. Disrupting the capsule, exposes the bacterial surface, rendering the cells more susceptible to immune attack. The *S. equi* subsp. *equi* Δ*hasA* (hyaluronan synthase) mutant is known to be highly susceptible to killing in equine blood, and so it was expected that this gene would be identified as being required for fitness in whole blood using TraDIS (Fig. 1a, Table 1) and in the validation experiment (Fig. 5). Interestingly, the log_2_FC for *hasA* determined in the TraDIS screen of −2.4 (*q=*0.046) was close to the threshold of −2 used to determine attenuation; yet in isolation, the Δ*hasA* mutant was dramatically reduced in fitness in whole equine blood. One explanation for this result is that acapsular mutants benefit from the retained capsule of neighbouring mutants during fitness studies. Such a bystander effect could explain the recovery of acapsular mutants from the guttural pouches of persistently infected horses [64]. Mutants in the gene encoding UDP-glucose 6-dehydrogenase, *hasB*, were also found to meet the fitness threshold in the whole blood TraDIS screen (log_2_FC=−2.2, *q=*<0.0005). However, the analysis of *hasC* was confounded by the presence of two copies of this gene in the genome of *Se*4047 [25]. Although important for fitness in whole blood, acapsular IS*S1* mutants in *hasA* had no fitness cost when exposed to H_2_O_2_ (log_2_FC=0.6, *q=*1). Our data suggest that at least some of the other 21 genes that were identified as being required for fitness in whole blood, but not H_2_O_2_, might similarly play a role in the evasion of phagocytosis, highlighting the application of TraDIS as a whole-genome functional genomics tool.

Other genes that were identified as being required for fitness in the presence of whole blood, but not H_2_O_2_, included *ccpA*, which putatively encodes catabolite control protein A. In *Streptococcus suis *, CcpA regulates many genes, primarily targeting those involved in carbohydrate metabolism and amino acid transporters, such as PTS uptake systems [65, 66]. Two PTS genes putatively required for mannose import, SEQ_0492 and SEQ_0494, and *ldh*, encoding lactate dehydrogenase, were also identified as important for survival in whole equine blood. Ldh was regulated by CcpA in *S. suis* [65, 66] and so the role of these genes in conferring fitness in the presence of whole equine blood may be interlinked.

The genes *pptAB* (also known as *ecsAB*) were required for the fitness of *S. equi* subsp. *equi* in whole blood, but not in the presence of H_2_O_2_. The *pptAB* genes encode ABC transporter proteins that export the quorum sensing peptides, SHP2 and SHP3, into the extracellular environment [67]. A *pptAB* deletion mutant of *Staphylococcus aureus* caused milder synovitis and reduced bone erosions in a murine model of arthritis [68]. The further study of *pptAB* to determine the role of quorum sensing to the virulence of *S. equi* subsp. *equi* is now warranted.

The transcriptional regulator CtsR was identified as being important for fitness in H_2_O_2_, but not whole equine blood. However, closer inspection of our data revealed that very few IS*S1* mutants existed in the three input libraries and that these were represented by few reads; in library AC, 2 mutants were represented by 14 reads, in CT 4 mutants were represented by 17 reads and in GA 2 mutants were represented by 22 reads (Table S6). Therefore, although this gene met our inclusion criteria, we believe that this hit may be a false positive. In support of this hypothesis, a Δ*ctsR* mutant of *Lactobacillus plantarum* was not significantly more susceptible to H_2_O_2_ than the wild-type parental strain [69].

With the exception of the capsule synthesis genes, none of the other genes that were previously linked to the survival of *S. equi* in whole blood were identified by TraDIS. The immunoglobulin-cleaving enzymes IdeE, IdeE2 [22, 23] and the Factor X-binding protein Se18.9 [21] are secreted and IS*S1*-associated defects are likely to be complemented by surrounding mutants. Unfortunately, the gene encoding the antiphagocytic protein SeM [19, 20] was removed from our analysis, as it was classed an essential gene in *Se*4047 [32]. None of the four fibronectin-binding proteins encoded by *S. equi* [24] were identified by TraDIS, which suggests that they may be functionally redundant in this system.

### Conclusions

The TraDIS screens described herein have identified some interesting and novel genes, with many of them commonly identified between conditions. Two of the genes included in the validation panel, *recG* and *addA,* were attenuated in both conditions, but exhibited a slow growth phenotype compared to the wild-type parental strain, which restricts their usefulness as future targets for the development of live-attenuated vaccines as, for manufacturing purposes, vaccine strains should not be attenuated for growth *in vitro*. The capsule mutant *hasA* was confirmed to be required for survival in whole equine blood, but not H_2_O_2_. However, the reduced fitness of *mnmE* mutants in the TraDIS screen was only recapitulated in H_2_O_2_. The *pyrP* mutant had a normal growth rate *in vitro* and a slower growth rate in whole equine blood, suggesting that the deletion of *pyrP* may be useful for the development of safer live attenuated vaccines. Therefore, further validation of the genes identified by TraDIS is warranted to demonstrate their importance in the absence of competing strains, prior to the development of new live attenuated vaccines.

## Data Bibliography

Charbonneau ARL, Taylor E, Mitchell CJ, Robinson C, Cain AK, Leigh JA, Maskell DJ, Waller AS.

Raw Illumina fastq files have been made available in Genbank at the Sequence Read Archive (SRA accession number: PRJNA578912). 2020.

## Supplementary Data

Supplementary material 1
