# Supplementary material for: Identification of genes required for the fitness of Streptococcus equi subsp. equi in whole equine blood and hydrogen peroxide
Source: Microb Genom. 2020 Mar 31;6(4):e000362. doi: 10.1099/mgen.0.000362 (PMC7276704; doi:10.1099/mgen.0.000362)
Supplement: Supplementary material 1 [file mgen-6-362-s001.pdf]

Supplementary Data

Table S1. Oligonucleotide primers used in this study. Restriction sites are underlined.

Primer name	Sequence (5’-3’)	Concentration
Adaptor primer 1Δ	P-GATCGGAAGAGCACACGTCT	100 µM
Adaptor primer 2Δ	ACACTCTTTCCCTACACGACGCTCTTCCGATC×T	100 µM
Specific IS*S1* primer	AATGATACGGCGACCACCGAGATCTACACGTTCATTGATATATCCTCGCTG	25 µM
Indexing primer AHT 6	CAAGCAGAAGACGGCATACGAGATCCTTACCATAACACTCTTTCCCTACACGACGCTCTTCCGATCT	25 µM
Indexing primer AHT 7	CAAGCAGAAGACGGCATACGAGATTGATATCTCTACACTCTTTCCCTACACGACGCTCTTCCGATCT	25 µM
Indexing primer AHT 15	CAAGCAGAAGACGGCATACGAGATGATAGAGACAACACTCTTTCCCTACACGACGCTCTTCCGATCT	25 µM
Indexing primer AHT 16	CAAGCAGAAGACGGCATACGAGATATCATAGACGACACTCTTTCCCTACACGACGCTCTTCCGATCT	25 µM
Indexing primer AHT 21	CAAGCAGAAGACGGCATACGAGATCGCTGCAGTAACACTCTTTCCCTACACGACGCTCTTCCGATCT	25 µM
Indexing primer AHT 32	CAAGCAGAAGACGGCATACGAGATTACACTCATGACACTCTTTCCCTACACGACGCTCTTCCGATCT	25 µM
Custom read 1 primer	GTTCATTGATATATCCTCGCTGTCATTTTTATTCATTTTACACTAAAATAGACTTAT	100 µM
Custom Index Read primer	AGATCGGAAGAGCGTCGTGTAGGGAAAGAGTGT	100 µM
P1 *mnmE*	GACACGAATTCCGTGGTTGAAAAAGAAGCC	100 µM
P2 *mnmE*	GACACGATATCCAATGCCAATAGCTCCTTCAC	100 µM
P3 *mnmE*	GACACGATATCCCTAGGAGAAATCACAGGCG	100 µM
P4 *mnmE*	GACACGTCGACCTTTGGACGCTTGCTTGAG	100 µM
P1 *pyrP*	GACACGAATTCCATGAAGCGTGCGATCAC	100 µM
P2 *pyrP*	GACACGATATCGCCTTTGGCACTTCTTCTAC	100 µM
P3 *pyrP*	GACACGATATCCAATGGCCTTCAGATTTCG	100 µM
P4 *pyrP*	GACACGTCGACCCTCCATTAACGATAGAGGC	100 µM
P1 *addA*	GACACGAATTCGCTTGAGTCCTCAGCTTGTGAC	100 µM
P2 *addA*	GACACGATATCCTCCTGCTGCAAACGAGC	100 µM
P3 *addA*	GACACGATATCGGGTGGATCACAGCTAGAAG	100 µM
P4 *addA*	GACACGTCGACCAGGAGAGCCTTCTATCCAG	100 µM
P1 *recG*	GACACGAATTCCTTCTAGACAAGCACCCTGCC	100 µM
P2 *recG*	GACACGATATCGACCCTTCAAATTAGCAATCG	100 µM
P3 *recG*	GACACGATATCGGCAAGACGAGTCGCTGCT	100 µM
P4 *recG*	GACACGTCGACGCTGAGCCAAGGGTTCGCTT	100 µM
5’9	CTGGAACATCTGTGGTATGG	100 µM
3’9	GCGTACCTTGGATATTCACC	100 µM

Table S2. Percent of sequencing reads mapping to the *S. equi* genome for each library. A stringent 100% match requirement was imposed on the data.

Library	% of sequence reads mapping to *S. equi* genome	
	Whole equine blood	H_2O_2
AC-IN	56.6	37.8
AC-OUT	52.8	38.5
CT-IN	54.8	40.3
CT-OUT	57.4	39.6
GA-IN	57.6	40.2
GA-OUT	55.7	44.8

Table S3. Composition of whole equine blood input and output libraries pre- and post-filtering.

Library	Unique insertion sites in genes	Total read count	Genes containing insertions (% of total genes : % of non-essential genes)
**AC-IN^{pre}**	30,181	875,825	1,768 (81.7 : 100)
**CT-IN^{pre}**	27,764	886,661	1,744 (80.6 : 100)
**GA-IN^{pre}**	32,011	761,827	1,760 (81.3 : 100)
**AC-IN^{post}**	26,381	769,660	1,503 (69.4 : 94.5)
**CT-IN^{post}**	24,353	770,366	1,503 (69.4 : 94.5)
**GA-IN^{post}**	28,128	770,193	1,503 (69.4 : 94.5)
**AC-OUT^{pre}**	24,985	689,276	1,676 (77.4 : 100)
**CT-OUT^{pre}**	25,593	744,624	1,666 (76.9 : 100)
**GA-OUT^{pre}**	30,404	721,703	1,687 (77.9 : 100)
**AC-OUT^{post}**	21,996	607,834	1,503 (69.4 : 94.5)
**CT-OUT^{post}**	22,555	607,834	1,503 (69.4 : 94.5)
**GA-OUT^{post}**	26,818	607,834	1,503 (69.4 : 94.5)

Table S4. Read counts for genes identified as important for *S. equi* fitness in whole equine blood. Read counts for each barcoded library before (IN) and after (OUT) exposure to whole equine blood are presented. Genes highlighted in grey were deleted by allelic replacement mutagenesis and deletion strains incubated in whole equine blood to validate the TraDIS results. An Δ*eqbE* deletion strain was used as a negative control.

Gene	Locus tag	AC-IN	AC-OUT	CT-IN	CT-OUT	GA-IN	GA-OUT
ackA	SEQ0118	54	12	66	3	52	4
SEQ0231	SEQ0231	1269	105	761	65	848	114
hasA	SEQ0269	156	26	249	34	634	84
hasB	SEQ0270	807	158	771	143	788	98
SEQ0306	SEQ0306	84	0	56	0	19	0
pepX	SEQ0383	295	61	313	31	322	51
recG	SEQ0454	440	33	170	7	199	9
SEQ0492	SEQ0492	72	7	22	0	49	4
SEQ0494	SEQ0494	41	0	28	0	46	6
pptA/ecsA	SEQ0506	123	15	87	0	111	8
pptB/ecsB	SEQ0507	355	41	330	29	248	34
SEQ0562	SEQ0562	105	7	109	13	72	13
bipA/typA	SEQ0615	77	4	42	0	13	0
pyrD	SEQ0655	85	10	83	5	64	6
ppc	SEQ0776	403	3	301	3	250	5
addA	SEQ0953	82	0	117	0	47	0
SEQ1028	SEQ1028	43	0	29	3	22	0
SEQ1073	SEQ1073	35	0	44	0	22	0
SEQ1112	SEQ1112	43	0	38	3	33	0
SEQ1146	SEQ1146	42	0	64	3	72	0
ldh	SEQ1169	36	0	76	3	64	0
SEQ1180	SEQ1180	56	0	44	3	16	0
SEQ1181	SEQ1181	73	0	21	0	19	0
SEQ1304	SEQ1304	348	3	215	3	229	0
pyrP	SEQ1316	77	0	127	6	113	4
mnmE	SEQ1365	127	4	53	0	31	0
SEQ1540	SEQ1540	100	3	115	5	16	0
smc	SEQ1566	439	40	265	10	484	15
ccpA	SEQ1596	47	3	28	0	11	0
pepQ	SEQ1597	79	0	59	0	165	5
SEQ1800	SEQ1800	56	0	49	0	33	0
scpA	SEQ1863	81	3	79	3	49	0
greA	SEQ1879	83	0	24	0	17	0
csrS	SEQ1889	180	3	34	0	82	0
yqeK	SEQ1909	56	4	29	0	41	0
pyrG	SEQ1945	809	133	652	102	903	118
eqbE	SEQ1242	6,463	6,483	7,903	8,716	4,690	5,778

Table S5. Composition of hydrogen peroxide input and output libraries pre- and post-filtering.

Library	Unique insertion sites in genes	Total read count	Genes containing insertions (% of total genes : % of non-essential genes)
**AC-IN^{pre}**	27,920	647,044	1,747 (80.7 : 100)
**CT-IN^{pre}**	25,951	648,236	1,720 (79.4 : 100)
**GA-IN^{pre}**	29,973	631,309	1,737 (80.2: 100)
**AC-IN^{post}**	24,372	567,560	1,471 (67.9 : 92.5)
**CT-IN^{post}**	22,734	569,090	1,471 (67.9 : 92.5)
**GA-IN^{post}**	26,226	568,628	1,471 (67.9 : 92.5)
**AC-OUT^{pre}**	25,239	718,526	1,680 (77.6: 100)
**CT-OUT^{pre}**	28,658	2,020,075	1,718 (79.4: 100)
**GA-OUT^{pre}**	27,436	551,893	1,674 (77.3 : 100)
**AC-OUT^{post}**	22,182	1,406,042	1,471 (67.9 : 92.5)
**CT-OUT^{post}**	25,138	1,406,042	1,471 (67.9 : 92.5)
**GA-OUT^{post}**	24,179	1,406,042	1,471 (67.9 : 92.5)

Table S6. Read counts for genes identified as important for *S. equi* fitness in Todd-Hewitt containing H_2O_2. Read counts for each barcoded library before (IN) and after (OUT) exposure to H_2O_2 are presented. One gene highlighted in blue was uniquely identified in the presence of H_2O_2 when compared to genes identified as reduced in fitness in whole equine blood. The remaining genes were similarly identified as required in whole equine blood. The genes highlighted in grey were deleted by allelic replacement mutagenesis and deletion strains incubated in Todd-Hewitt containing H_2O_2 to validate TraDIS results. The Δ*hasA*, Δ*pyrP* and Δ*eqbE* deletion strains were used as negative controls in the validation experiments.

Gene	Locus tag	AC-IN	AC-OUT	CT-IN	CT-OUT	GA-IN	GA-OUT
SEQ0118	SEQ0118	59	7	69	13	25	0
ctsR	SEQ0200	14	0	17	8	22	0
SEQ0306	SEQ0306	35	0	30	7	31	0
recG	SEQ0454	233	9	99	22	130	0
SEQ0562	SEQ0562	57	0	69	0	38	0
ppc	SEQ0776	292	65	206	35	123	0
addA	SEQ0953	54	0	71	3	52	0
SEQ1028	SEQ1028	33	7	21	4	13	0
SEQ1146	SEQ1146	35	0	33	0	13	0
ldh	SEQ1169	24	0	36	9	35	0
SEQ1304	SEQ1304	259	7	128	18	186	0
mnmE	SEQ1365	65	7	35	8	40	0
smc	SEQ1566	262	51	197	71	349	31
pepQ	SEQ1597	72	7	83	7	99	12
yqeK	SEQ1909	42	7	15	3	45	0
hasA	SEQ0269	126	292	128	573	663	2,455
pyrP	SEQ1316	23	33	82	150	68	65
eqbE	SEQ1242	4,397	15,062	6,294	19,305	3,429	11,547

Table S7. *S. equi* genes with reduced fitness following overnight growth in THB as a result of IS*S1* insertion as identified by TraDIS. Genes highlighted in grey were deleted by allelic replacement mutagenesis based on the whole blood and H_2O_2 fitness data. An Δ*eqbE* deletion strain was used as a negative control.

Gene	Locus tag	Function	Log_2FC	*q* value
pyrD	SEQ0655	putative dihydroorotate dehydrogenase	-4.4	0.002
sufB	SEQ1926	FeS assembly protein	-2.6	0.002
sufD	SEQ1929	FeS assembly protein	-2.5	0.032
SEQ1930	SEQ1930	putative ABC transporter, ATP-binding protein	-1.8	-0.022
SEQ2142	SEQ2142	putative anaerobic ribonucleoside-triphosphate reductase activating protein	-3.3	0.002
SEQ2146	SEQ2146	anaerobic ribonucleoside-triphosphate reductase	-3.0	0.0001
hasA	SEQ0269	hyaluronan synthase	-1.8	1
recG	SEQ0454	ATP-dependent DNA helicase	-0.7	1
addA	SEQ0953	putative ATP-dependent exonuclease subunit A	-2.0	1
eqbE	SEQ1242	equibactin nonribosomal peptide synthase protein	0.3	1
pyrP	SEQ1316	uracil permease	-0.3	1
mnmE	SEQ1365	tRNA modification GTPase	-0.2	1
